# BVES is a novel interactor of ANO5 and regulates myoblast differentiation

**DOI:** 10.1186/s13578-021-00735-w

**Published:** 2021-12-28

**Authors:** Haiwen Li, Li Xu, Yandi Gao, Yuanbojiao Zuo, Zuocheng Yang, Lingling Zhao, Zhiheng Chen, Shuliang Guo, Renzhi Han

**Affiliations:** 1 Division of Cardiac Surgery, Department of Surgery, Davis Heart and Lung Research Institute, The Ohio State University Wexner Medical Center, Columbus, OH 43210 USA; 2 Department of Pediatrics, The Third Xiangya Hospital, Central South University, Changsha, China

**Keywords:** ANO5, BioID2, BVES, Muscle differentiation, Muscular dystrophy, Proximity labeling

## Abstract

**Background:**

Anoctamin 5 (ANO5) is a membrane protein belonging to the TMEM16/Anoctamin family and its deficiency leads to the development of limb girdle muscular dystrophy R12 (LGMDR12). However, little has been known about the interactome of ANO5 and its cellular functions.

**Results:**

In this study, we exploited a proximal labeling approach to identify the interacting proteins of ANO5 in C2C12 myoblasts stably expressing ANO5 tagged with BioID2. Mass spectrometry identified 41 unique proteins including BVES and POPDC3 specifically from ANO5-BioID2 samples, but not from BioID2 fused with ANO6 or MG53. The interaction between ANO5 and BVES was further confirmed by co-immunoprecipitation (Co-IP), and the N-terminus of ANO5 mediated the interaction with the C-terminus of BVES. ANO5 and BVES were co-localized in muscle cells and enriched at the endoplasmic reticulum (ER) membrane. Genome editing-mediated ANO5 or BVES disruption significantly suppressed C2C12 myoblast differentiation with little impact on proliferation.

**Conclusions:**

Taken together, these data suggest that BVES is a novel interacting protein of ANO5, involved in regulation of muscle differentiation.

**Supplementary Information:**

The online version contains supplementary material available at 10.1186/s13578-021-00735-w.

## Introduction

ANO5 is a member of the TMEM16/Anoctamin family with putative chloride channel function and/or phospholipid scrambling activity [1–3]. Genetic mutations in the *ANO5* gene cause recessive LGMDR12 and Miyoshi myopathy type 3 (MMD3) characterized by progressive muscle weakness and degeneration, and dominant gnathodiaphyseal dysplasia (GDD) [4]. Ano5 deficient mice exhibited little pathology in skeletal muscle [5, 6] while others reported a mild muscular dystrophy phenotype in a different strain of *Ano5*-KO mice, which also exhibited defective myoblast fusion and delayed muscle regeneration after cardiotoxin injection [7]. In line with a potential role of ANO5 in muscle regeneration, the LGMDR12 patients manifested clinical symptoms that persisted longer than normal after a significant muscle injury [8]. Moreover, several recent studies revealed that Ano5 may participate in plasma membrane repair (PMR) through coordination with other membrane repair proteins such as dysferlin, annexin A1-A6, or regulating sarcoplasmic reticulum Ca^2+^ [7, 9–11]. Thus, defective PMR may also contribute to the development of muscular dystrophy caused by *ANO5* mutations.

Defining specific ANO5 interactions and spatially or temporally restricted local proteomes would potentially improve our understanding of ANO5-regulated cellular processes. Recent technological advancements in proximity labeling with mutant biotin ligase variants such as BioID, BioID2, TurboID enable discovery of protein neighborhoods defining functional protein complexes and/or organelle compositions. Here we employed BioID2 to depict the Ano5 interactome and identified a novel Ano5-interacting protein, BVES (Blood Vessel Epicardial Substrance, also known as POPDC1), which is highly expressed in striated muscles. Interestingly, genetic mutations in BVES are linked to LGMDR25 and cardiac arrhythmia [12–17], suggesting that LGMDR12 and LGMDR25 may share some common pathogenic mechanisms.

## Results

### BioID2 proximity labeling of ANO5-interacting proteins in C2C12 muscle cells

**Fig. 1:**
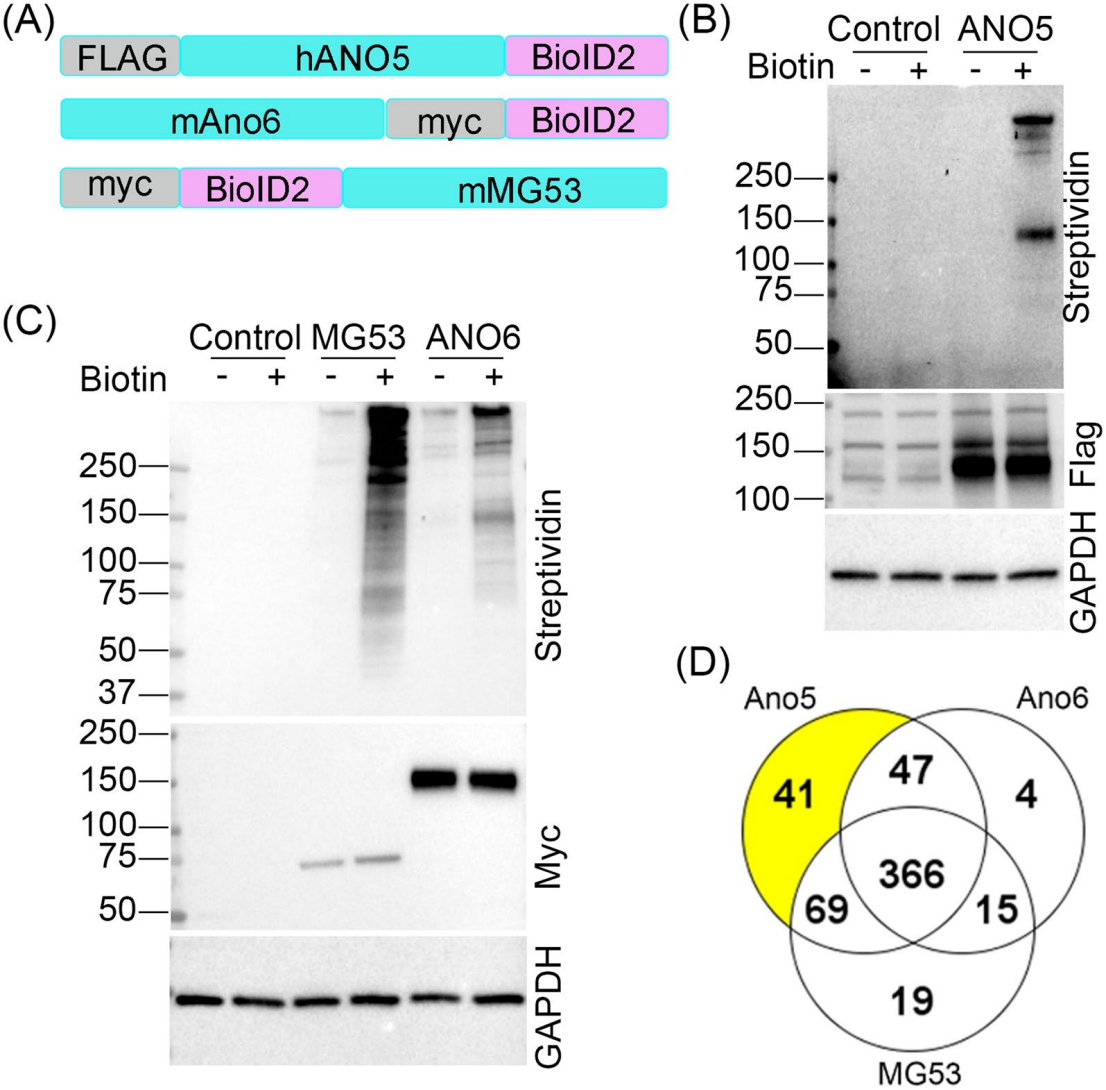
Identification of ANO5 interactome in C2C12 muscle cells by BioID2 proximal labeling. **A** Schematic maps of the BioID2 fusion constructs with hANO5, mMG53 or mAno6. **B**, **C** Western blot analysis of C2C12 stably expressing hANO5 (**B**), mMG53 and mAno6 (**C**) fused with BioID2. GAPDH was used as a loading control. **D** Venn diagram of the total number of proteins identified for each fusion protein: hAno5-BioID2, BioID2-mMG53, and mAno6-BioID2

We employed proximity labeling with Bid-ID2 to identify ANO5-interacting proteins in C2C12 muscle cells as this approach has the advantage to capture weak/transient interactions with high sensitivity and low background contamination [18]. To increase experimental stringency, we used ANO6-BioID2 (an Ano5 paralogue) and BioID2-MG53 (a known PMR protein) as controls. C2C12 cells stably expressing ANO5-BioID2, ANO6-BioID2 or BioID2-MG53 with FLAG or myc tag (Fig. 1A) were generated by lentiviral transduction. Western blot showed that all three transgenes were expressed well in their corresponding C2C12 stable cell lines (Fig. 1B, C). To examine whether the BioID2 in the fusion proteins can biotinylate endogenous proteins, the stable cell lines were analyzed by Western blot using streptavidin-HRP after incubation with or without 50 µM biotin for 24 h. As expected, the biotinylated proteins were readily detectable in the lysates from the stable ANO5-BioID2, ANO6-BioID2 and BioID2-MG53 C2C12 cells with biotin incubation as compared to the cells without biotin treatment, which showed minimal background biotinylation similar to the control C2C12 sample (Fig. 1B, C).

The biotinylated proteins were isolated using streptavidin magnetic beads and subjected to mass spectrometry for protein identification. A total of 561 unique proteins with a minimum of 2 unique peptides and a false discovery rate (FDR) below 1.0% were identified from the three samples with 366 shared among them (Fig. 1D). Forty-one unique proteins were specifically identified in the ANO5-BioID2 sample (Fig. 1D, Additional file 1: Table S1).

### Validation of BVES-ANO5 interaction

**Fig. 2:**
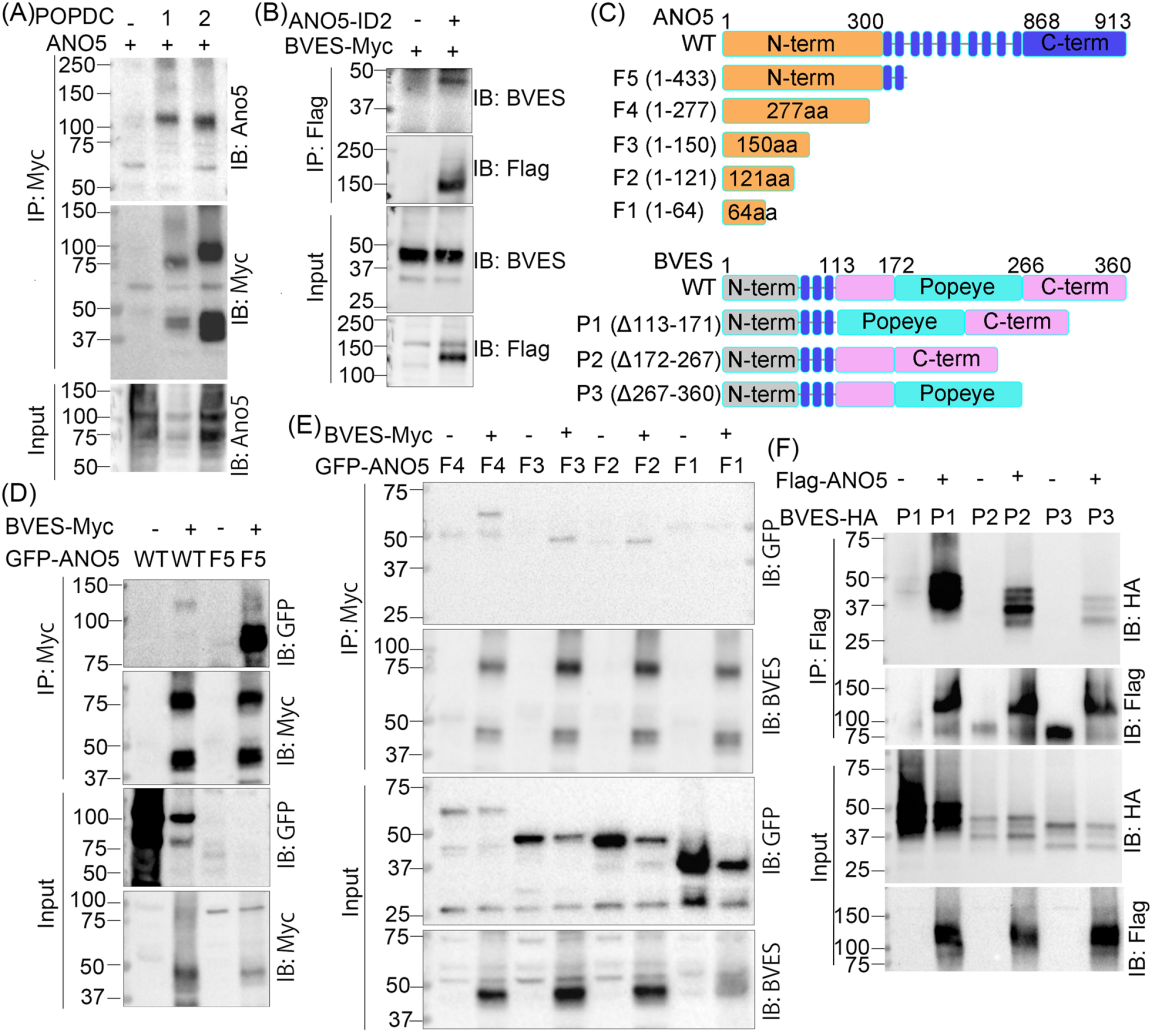
Validation of BVES-ANO5 interaction by Co-IP. **A** ANO5 was co-immunoprecipitated with BVES-myc and POPDC2-myc using the myc antibody from the lysates of Cos-1 cells with co-expression of ANO5 and BVES or POPDC2. **B** BVES was co-immunoprecipitated with FLAG-hANO5-BioID2 using the FLAG antibody from the lysates of C2C12 with co-expression of FLAG-hANO5-BioID2 and BVES-myc. **C** Schematic view of the mutant ANO5 and BVES constructs. **D** BVES-myc efficiently pulled down the N-terminal of Ano5. **E** Mapping the N-terminal ANO5 fragments interacting with BVES. **F** Mapping the BVES domains interacting with ANO5

Among the unique proteins identified in the ANO5-BioID2 sample, two members of the three POPDC family proteins including BVES and POPDC3 were of particular interest for the following reasons. Both genes were highly expressed in striated muscles [19–21] and their genetic mutations were linked to LGMD patients with or without cardiac involvement [22]. To validate the interaction between ANO5 and BVES, we performed Co-IP in COS-1 cells co-expressing ANO5 and myc-tagged BVES, POPDC2 or POPDC3. Preliminary experiment found that POPDC3 was poorly expressed in COS-1 cells (Additional file 1: Fig. S1) and thus it was not included in the final Co-IP experiment. As shown in Fig. 2A, ANO5 was specifically pulled down with BVES and POPDC2 by the anti-myc antibody. Similarly, this interaction also occurred in C2C12 myoblasts stably expressing ANO5-BioID2 transiently transfected with BVES-myc (Fig. 2B). We generated a series of truncations or deletions to map the interaction domains between ANO5 and BVES (Fig. 2C). Both full-length and the N terminal fragment of ANO5 were readily pulled down by BVES-myc (Fig. 2D). We further narrowed down the first 121 amino acids of ANO5 mediating the ANO5-BVES interaction (Fig. 2E). Similarly, the Co-IP studies with BVES deletion or truncation mutants showed that the C-terminal region of BVES was involved in mediating the ANO5-BVES interaction. These biochemical data confirmed that BVES is a novel ANO5-interacting protein.

### Co-localization of ANO5 and BVES in muscle and non-muscle cells

**Fig. 3:**
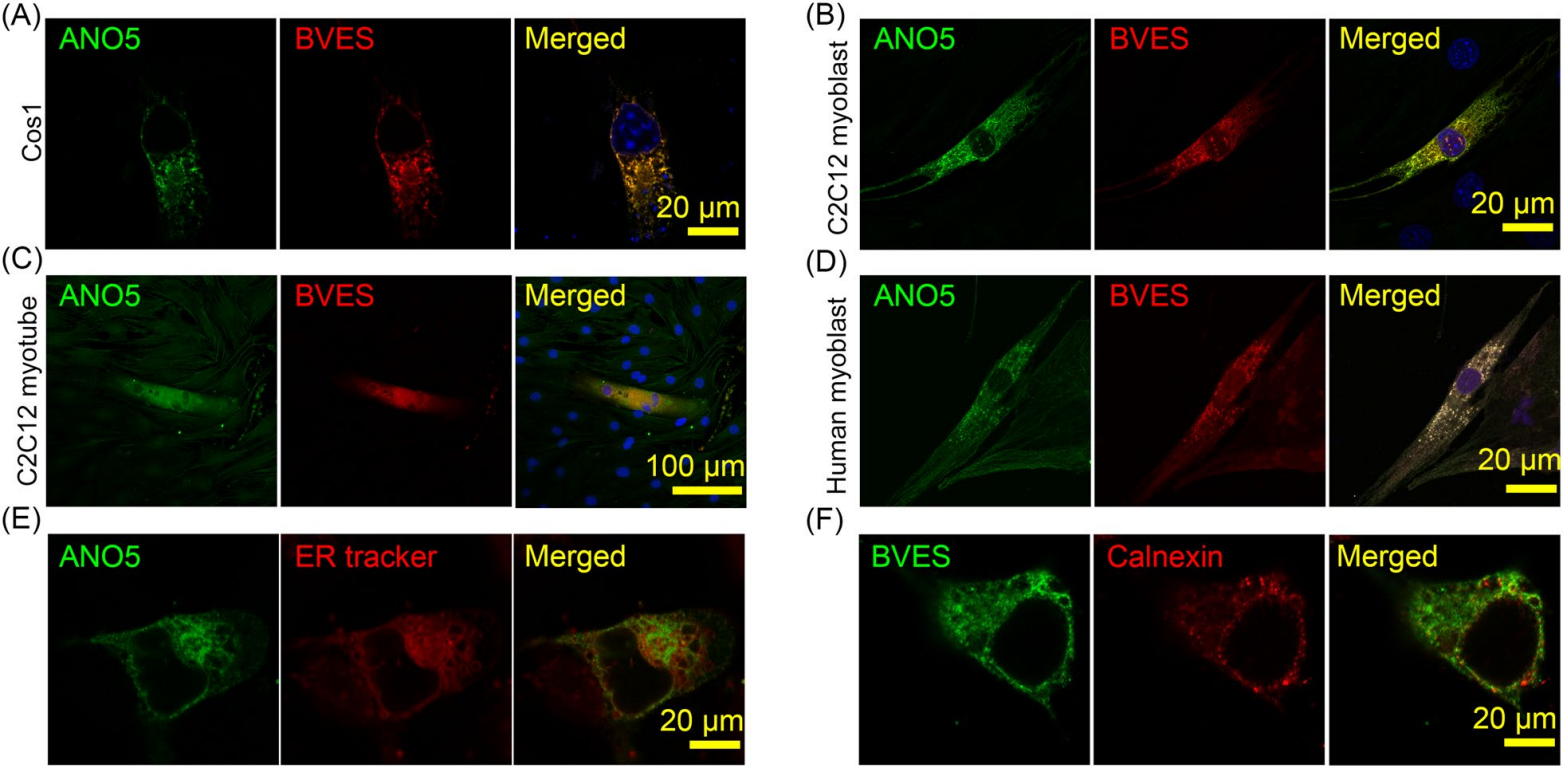
Subcellular localization of ANO5 and BVES by confocal imaging. **A**–**C** Confocal imaging of Cos-1 (**A**), C2C12 myoblasts (**B**) or myotubes (**C**) co-expressing BVES-myc and GFP-ANO5. **D** Confocal imaging of human myoblasts co-expressing BVES-myc and mCherry-ANO5. **E** Live cell imaging of Cos-1 cells expressing GFP-ANO5 with ER-Tracker Red. **F** Immunofluorescence imaging of Cos-1 cells expressing BVES-myc, stained with with antibodies against myc and calnexin

We performed immunofluorescence staining and confocal imaging to analyze the cellular localization of ANO5 and BVES. Substantial co-localization of ANO5 and BVES were observed in COS-1 cells (Fig. 3A), C2C12 myoblasts (Fig. 3B), C2C12 myotubes (Fig. 3C) and human myoblasts (Fig. 3D) co-expressing these two proteins. Consistent with previous studies showing that ANO5 was enriched at the ER membrane [23, 24], we observed that there was substantial fluorescence overlapping between GFP-ANO5 and ER tracker (Fig. 3E). Immunofluorescence staining also showed that BVES-myc was co-localized with the ER marker calnexin (Fig. 3F). In addition, we performed immunofluorescence staining of BVES and other subcellular markers. BVES was also found to be co-localized with the early endosome marker EEA1, but not with the markers for late endosome (Rab7), recycling endosome (Rab11) or lysosome (LAMP1) (Additional file 1: Fig. S2).

### Generation of *Ano5*- and *Bves*-KO C2C12 cells by genome editing

**Fig. 4:**
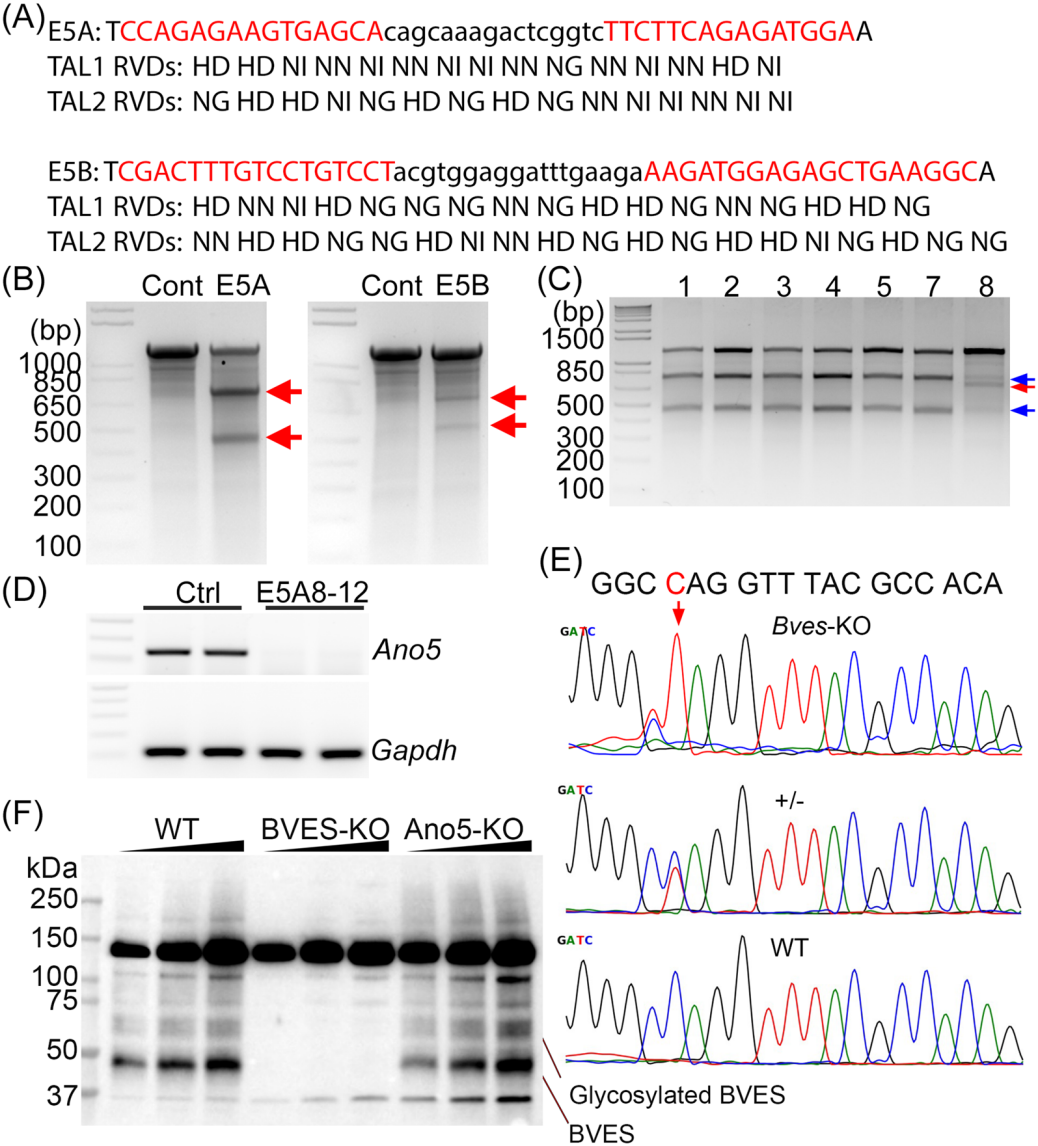
Generation of Ano5-KO and BVES-KO C2C12 cells by gene editing. **A** Design of two TALEN pairs (E5A and E5B) targeting the exon 5 of mouse *Ano5*. The left and the right target sites were colored in red, with the repeat-variable di-residues (RVD) of each TAL shown below. **B** T7E1 assay of the gene editing activity conferred by E5A and E5B TALEN pairs in C2C12 myoblasts. The red arrows indicate the T7E1 enzyme cleavage bands shown in TALEN-transfected cells but not in control (Ctrl) samples. **C** Screening of individual C2C12 clones transfected with E5A TALEN pair by T7E1 assay. The blue arrows indicate the predicted T7E1 cleavage products. The red arrow indicates a smaller product appeared in Clone 8. **D** RT-PCR analysis of *Ano5* expression in control and Clone E5A8-12. **E** Generation of *Bves*-KO C2C12 cells by CBE base editing. The gRNA targeting the exon 4 of mouse *Bves* was designed to install a premature TAG stop codon at position 159 (highlighted in red). Sanger sequencing confirmed the genotype of BVES-KO C2C12 cells. **F** Western blot analysis of BVES expression in the total membrane fractions of WT, BVES-KO and Ano5-KO C2C12 myoblasts

Transcription activator like effector nuclease (TALEN)-mediated genome editing was used to generate *Ano5*-KO C2C12 cells. Two TALEN target sites were chosen from the exon 5 of mouse *Ano5* gene and two pairs of TALENs (Fig. 4A) were assembled using the Golden-gate TALEN kit as described before [25]. The TALEN plasmids were transfected into C2C12 cells and genome editing was verified by T7E1 assay (Fig. 4B). As the E5A-TALEN pair appeared to have a higher editing efficiency than the E5B-TALEN pair, we used the E5A-TALEN pair transfected C2C12 cells for single cell colony derivation. Forty-eight hours after transfection with the E5A-TALEN pair, the C2C12 cells were serially diluted for single cell clone screening. Individual clones were randomly picked up and screened by PCR and T7E1 assay. The clone 8 showed additional lower band besides the two cleavage bands of expected sizes from T7E1 digestion (Fig. 4C), and this additional band was found to carry a large 542-bp deletion around the target site (Additional file 1: Fig. S3). The Clone 8 was chosen for further purification by serial dilutions. Twelve clones were then screened for the deletion PCR product and differentiation ability. The E5A8-12 clone was found to carry homozygous 542-bp deletion, which is expected to disrupt the reading frame of *Ano5*. This cell clone (designated as the *Ano5*-KO) can be differentiated to form myotubes (Additional file 1: Fig. S4). RT-PCR analysis confirmed that the *Ano5* transcript expression was disrupted in the Ano5-KO C2C12 cells following differentiation (Fig. 4D).

We similarly established *Bves*-KO C2C12 by using the state-of-the-art cytosine base editing (CBE) [26]. A premature stop codon was engineered into the coding exon 4 of *Bves* by transfection of C2C12 cells with CBE and a targeting gRNA (Fig. 4E). After cell sorting, a single cell clone was confirmed to carry the homozygous Q159X mutation by Sanger sequencing (Fig. 4E). This cell clone (designated as *Bves*-KO) can be differentiated to form myotubes (Additional file 1: Fig. S5). The loss of BVES expression was confirmed in *Bves*-KO C2C12 myotubes by Western blot (Fig. 4F). Multiple BVES specific bands at around 41 kDa and 60 kDa appeared in WT myotubes, corresponding to the monomer and glycosylated form, and these bands were disrupted in *Bves*-KO myotubes. Disruption of *Ano5* did not seem to alter the expression of BVES (Fig. 4F).

### Impact of ANO5 or BVES deficiency on C2C12 myoblast proliferation and differentiation

**Fig. 5:**
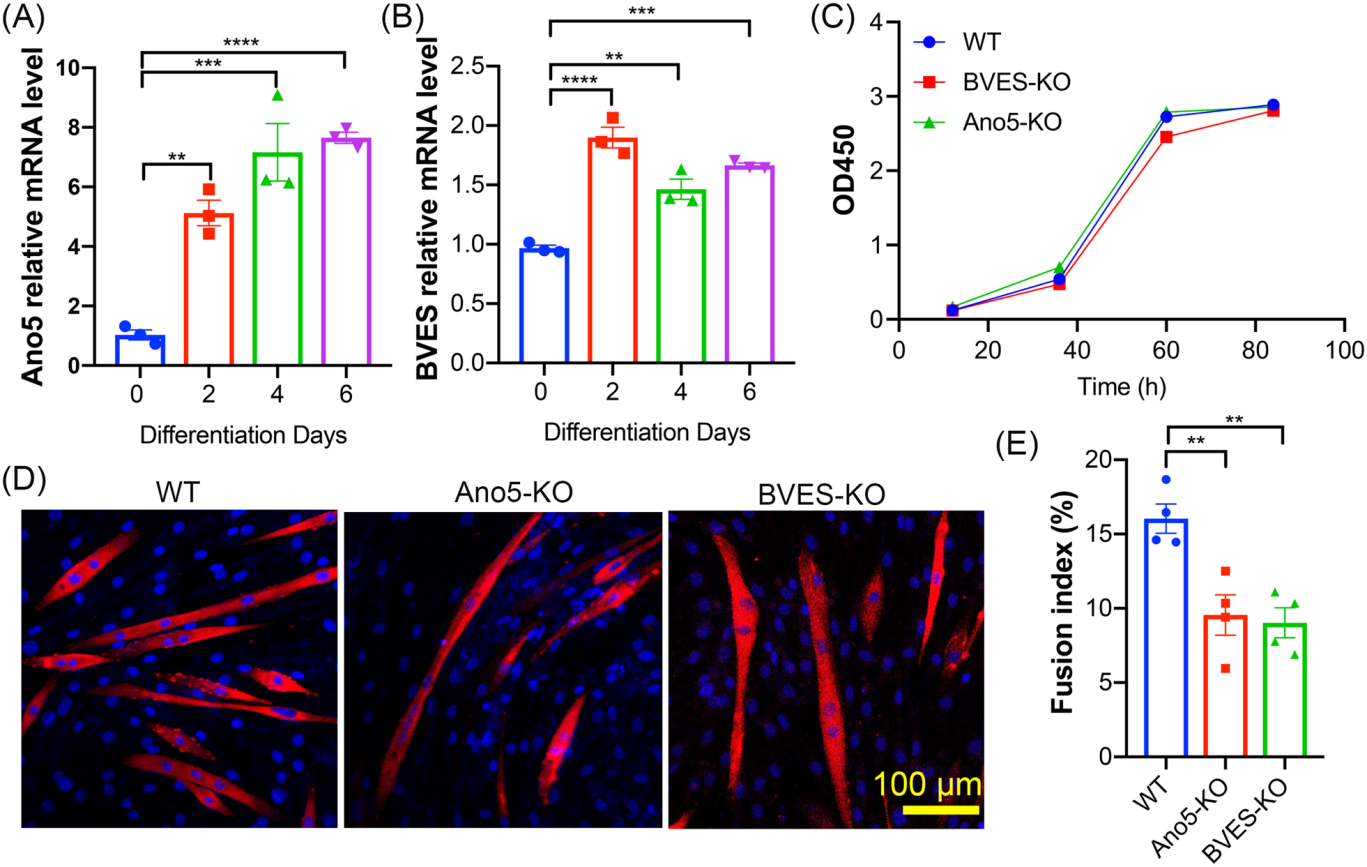
Impact of BVES or ANO5 deficiency on myoblast proliferation and differentiation. **A**, **B** Quantitative RT-PCR analysis of Ano5 (**A**) and BVES (**B**) expression during C2C12 myoblast differentiation. **p < 0.01, ***p < 0.001, ****p < 0.0001 (one-way ANOVA with Turkey’s post tests). **C** The CCK8 proliferation assay of WT, BVES-KO and Ano5-KO C2C12 myoblasts from 12 to 84 h in culture. **D** Immunofluorescence images of WT, BVES-KO and Ano5-KO C2C12 myotubes on day 4 of differentiation stained with the monoclonal antibody against myosin heavy chain (MHC) and DAPI. Scale bar: 100 µm. **E** Quantification of the fusion index of WT, BVES-KO and Ano5-KO C2C12 myotubes in differentiation medium for four days. **p < 0.01 (one-way ANOVA with Turkey’s post tests)

The expression of *Ano5* and *Bves* during C2C12 myoblast differentiation was examined by quantitative RT-PCR. As shown in Fig. 5A and B, the expression of both *Ano5* and *Bves* were significantly increased during myoblast differentiation, suggesting that they may regulate myogenesis. The CCK8 proliferation assay showed that *Ano5* or *Bves* gene disruption did not significantly affect myoblast proliferation (Fig. 5C). To examine the impact of *Ano5* or *Bves* gene disruption on myoblast differentiation, the WT, *Ano5*-KO and *Bves*-KO C2C12 cells were differentiated for 4 days and stained with myosin heavy chain (MyHC) and DAPI (Fig. 5D). The fusion index (the percentage of myonuclei in MyHC + cells with ≥ 2 nuclei/cell) was significantly decreased in *Ano5*-KO and *Bves*-KO cells as compared to the control cells (Fig. 5E), indicating that both ANO5 and BVES are indispensable for muscle differentiation.

## Discussion

In this study, we identified BVES as a new ANO5-interacting protein in skeletal muscle cells using the BioID2 proximity labeling approach and mapped the interacting domains between BVES and ANO5. We further showed that genetic disruption of *Bves* or *Ano5* with gene editing compromised C2C12 myoblast differentiation.

BVES is one of the three POPDC proteins encoded in mammalian genome. All *POPDC* genes are highly expressed in striated muscles [20]. While *BVES* expressed at similar levels in both skeletal muscle and heart, *POPDC3* is more selectively expressed in skeletal muscle and *POPDC2* is more restricted to heart [27]. Consistent with their tissue expression profiles, patients that carry mutations in *BVES* develop LGMDR25 and AV-block of varying degree [12–17], whereas mutations in *POPDC3* cause LGMD without affecting the heart [28] and the opposite is true for *POPDC2* [29]. All three POPDC proteins are believed to act as cAMP effectors because they all carry a high-affinity cAMP binding Popeye domain [30]. Previous studies showed that the POPDC proteins regulate the mechano-gated potassium channel TREK-1 in a cAMP-dependent manner [28, 30, 31]. However, it is unknown whether the alterations of the TREK-1 channel activity is directly responsible for muscular dystrophy and cardiac arrhythmia in the patients.

The physical interaction between ANO5 and BVES revealed in our study suggests that these proteins may participate in common cellular processes. Indeed, we showed that genetic disruption of *Ano5* or *Bves* impaired myoblast differentiation in C2C12 cells. In addition to regulating muscle differentiation, several recent studies demonstrated a role of ANO5 in PMR by regulating the SR calcium dynamics and coordinating with other PMR proteins [7, 9–11]. This raises an interesting possibility that BVES may also regulate PMR. If so, the cAMP signaling would very likely also be involved in the regulation of PMR. In supporting of this notion, *BVES* mutant LGMD patient muscle fibers showed the presence of plasma membrane discontinuities and submembraneous vacuoles as revealed by transmission electron microscopy analysis [31]. As an important second messenger, cAMP has been implicated in many aspects of muscle physiology, such as glycogenolysis, contractility, sarcoplasmic calcium dynamics, and hypertrophy [32]. Interestingly, the cAMP-PKA signaling was found to activate ANO9’s cation channel activity [33]. It is thus plausible to propose that BVES, as a cAMP effector protein, is involved in regulating ANO5’s channel and lipid scrambling activities [34, 35], thus facilitating proper execution of PMR, muscle differentiation and other unknown physiological processes.

In C2C12 cells, BVES was found to be mainly localized to the internal membrane structures such as ER and early endosomes, whereas previous studies showed clear plasma membrane localization in isolated adult cardiac myocytes and human skeletal muscle [31, 36]. It is not clear what mechanism regulates the different cellular localization of BVES in C2C12 muscle cells versus adult muscle fibers. One possibility is the glycosylation status of BVES as protein glycosylation plays an important role in plasma membrane localization of transmembrane proteins. We found that BVES ran into 50–70 KDa on SDS-PAGE in addition to the 42 kDa core peptide, consistent with previous reports that the Asn20 and Asn27 of BVES were glycosylated [37, 38]. Understanding the role of BVES glycosylation in muscle biology warrants future investigation.

## Conclusion

In conclusion, we showed that BVES is a novel ANO5-interacting protein, potentially participating in regulating common processes such as muscle differentiation and PMR. Future studies are required to dissect the molecular mechanisms for ANO5-BVES interaction in muscle biology and disease.

## Materials and methods

### Plasmid construction

The BioID2 was amplified by PCR from the Myc-BioID2-MCS plasmid (Addgene #74,223, Watertown, MA), fused with human *ANO5*, mouse *Ano6* or mouse *Mg53* cDNA, and inserted into pLVX-puro (Clontech, San Jose, CA) to obtain pLVX-hANO5-BioID2, pLVX-mAno6-BioID2, and pLVX-BioID2-mMG53, respectively. BVES-myc, POPDC2-myc and POPDC3 were constructed by inserting the corresponding cDNA into pCDNA3.1-myc backbone. ANO5 related plasmids were described previously [39]. The truncation or deletion mutant BVES constructs were generated by overlapping PCR. The Ano5-targeting TALEN pairs were assembled using the Golden Gate TALEN kit (Addgene #1000000016) as previously described [25] and the TALEN pairs were coupled with 2A peptide [40]. The protein sequences of Ano5-TALENs are provided in the Additional file 1: Fig. S6. pCMV-AncBE4max plasmid was obtained from Addgene (#112094). The pCMV-AncBE4-GFP was generated by ligation of SacI-EcoR1 fragment from pCMV-AncBE4max and the EcoRI/AgeI-digested GFP fragment into SacI/AgeI linearized pCMV-AncBE4max backbone. The annealed gRNA oligos (targeting mouse *Bves*) were cloned into pLenti-OgRNA-Zeo plasmid as previously described [41, 42]. All plasmids used in this study are listed in Additional file 1: Table S2.

### Cell culture and transfection

HEK293 and COS-1 cells were cultured in Dulbecco’s modified Eagle medium (DMEM, GIBCO) containing 1 g/l glucose and supplemented with 10% (v/v) fetal bovine serum (FBS, Sigma) and 1% (v/v) penicillin–streptomycin (Pen-Strep, Sigma-Aldrich). C2C12 and human myoblasts were grown in DMEM with 20% FBS and differentiated in DMEM with 2% horse serum after 70–80% confluency. All cells were cultured in a 37 °C incubator with a humidified 5% CO_2_ atmosphere. Transfection of HEK293 or COS-1 cells were performed using X-tremeGENE™ HP DNA transfection reagent (#6366244001, Sigma-Aldrich, St. Louis, MO). For C2C12 and human myoblasts, plasmids were transfected with the Neon electroporation system (Thermo Scientific, NY). For confocal imaging, the aforementioned cells were plated onto collagen-coated 35-mm glass-bottom dishes post transfection and grown for 24–48 h.

### Generation of *Bves*-KO and *Ano5*-KO C2C12 cells

To generate Ano5-KO C2C12 cells, the C2C12 cells were electroporated with pTAL9-Ano5E5A and individual cell clones were screened by PCR and T7E1 assay. To generate Bves-KO C2C12 cell line, the C2C12 cells were sorted for GFP into single cells in a 96-well plate after electroporation with the pCMV-AncBE4-GFP and pLenti-Bves-gRNA-Zeo. The expanded individual cell clones were screened by PCR and Sanger sequencing.

### BioID2 pull-down

For BioID2 pull down, C2C12 myotubes stably expressing hANO5-BioID2, mAno6-BioID2 or BioID2-mMG53 were incubated with 50 μM biotin for 16 h. After washing the cells with PBS twice very gently, the cells were lysed in 2.4 ml lysis buffer (50 mM Tris, pH 7.4, 500 mM NaCl, 0.4% SDS, 1 mM dithiothreitol, and 1 × complete protease inhibitor). Triton X-100 was added to 2% final concentration. After sonication, an equal volume of 50 mM Tris (pH 7.4) was added. After centrifugation at 16,500 × g for 10 min, the supernatant was collected to a 15-mL tube and incubated with 300 ul magnetic streptavidin beads (#88,816, Thermo Scientific, NY) overnight at 4 °C. Beads were washed according to the following steps: twice with 2% SDS, once with wash buffer containing 0.1% deoxycholate, 1% Triton X-100, 500 mM NaCl, 1 mM EDTA, and 50 mM HEPES, pH 7.5, once with wash buffer containing 250 mM LiCl, 0.5% NP-40, 0.5% deoxycholate, 1 mM EDTA, and 10 mM Tris, pH 8, and once with 50 mM Tris pH 8. Ten percent of the samples were saved for Western blot analysis. The other 90% of the samples were subjected to mass spectrometry analysis at the Ohio State University Comprehensive Cancer Center Proteomic Shared Resources.

### Cell proliferation assay

The cell proliferation was examined by the CCK-8 cell counting kit (Dojindo Molecular Technologies, MD, USA). WT, BVES-KO or Ano5-KO C2C12 cells were prepared in 96-well plates at the initial density of 5 × 10^3^ cells/well. The 450 nm absorbance was measured at 12–84 h according to the manufacturer’s instructions.

### Western blot

The cells were lysed with cold RIPA buffer supplemented with protease inhibitors, and the extracted proteins were quantified by DC™ Protein Assay Reagent (Bio-Rad Laboratories, Hercules, CA). The extracted protein samples were separated by stain-free SDS-PAGE gels (Bio-Rad Laboratories, 4–15%) and transferred onto Nitrocellulose Membranes (0.45 μm). Primary antibodies include the rabbit polyclonal anti-BVES (1:1000, HPA014788, Sigma-Aldrich, St. Louis, MO), mouse monoclonal anti-Ano5 (1:1000, N421A/85, UC Davis/NIH NeuroMab Facility, Davis, CA), anti-GAPDH (1:4000, MAB374, Cell Signaling Technology, Danvers, MA), anti-GFP antibody (1:1000, A01388, Genscript, Piscataway, NJ), streptavidin-HRP (1:20, DY998, R&D Systems, Minneapolis, MN), myc (1:1000, #2276, Cell Signaling Technology, Danvers, MA), FLAG (1:1000, #F3165, Sigma-Aldrich, St. Louis, MO) and HA (1:1000, #3724, Cell Signaling Technology, Danvers, MA). Secondary HRP-conjugated goat anti-mouse (1:4000), goat anti-rabbit (1:4000) antibodies were obtained from Cell Signaling Technology. The membranes were developed using ECL western blotting substrate (Pierce Biotechnology, Rockford, IL) and images were scanned with the ChemiDoc XRS + system (Bio-Rad Laboratories). Western blots were quantified using Image Lab 6.0.1 software (Bio-Rad Laboratories) according to the manufacturer’s instructions.

### Immunofluorescence staining

Cells were fixed with 4% paraformaldehyde for 10 min at room temperature. After washing with PBS, the slides were blocked with 5% BSA with 0.3% Triton X-100 for 1 h. The slides were incubated with primary antibodies at 4 °C overnight. The primary antibodies include myc (1:200, #2276, Cell Signaling Technology, Danvers, MA), calnexin (1:200, #2679, Cell Signaling Technology, Danvers, MA), and myosin heavy chain (1:200, MF20, Developmental Studies Hybridoma Bank, Iowa City, IA). The cells were then washed extensively with PBS and incubated with Alexa Fluor 568 (donkey anti-rabbit IgG, Invitrogen) or Alexa Fluor 488 (goat anti-rabbit IgG, Invitrogen) for 1 h at room temperature. The stained cells were sealed with VECTASHIELD Antifade Mounting Medium with DAPI (Vector Laboratory, Burlingame, CA). ER-tracker red (E34250, Thermo Scientific) was used for labeling endoplasmic reticulum in COS-1 cells after 24 h transfection with GFP-Ano5. All images were taken with a Zeiss 780 confocal microscope (Jena, Germany).

### Co-immunoprecipitation assay

Cells were transfected with the indicated plasmids and lysed in RIPA lysis buffer [25 mM Tris–HCl (pH 7.4), 150 mM NaCl, 5% Glycerol, 1% Triton X-100, 2 mM EDTA, and 1 mM DTT supplemented with protease inhibitor cocktail (Roche)] at 48 h after transfection. Immunoprecipitation was performed by incubation with the indicated primary antibodies for 4 h and protein A/G agarose beads (#20423, Thermo Scientific) overnight at 4 °C. The beads were washed at least three times with RIPA lysis buffer. Lysates and immunoprecipitated sampless were examined by using the indicated primary antibodies followed by the related secondary antibodies and the SuperSignal Chemiluminescence Kit (Thermo Fisher Scientific, Waltham, MA, USA).

### RNA extraction and quantitative RT-PCR analysis

Total RNA was extracted from C2C12 myoblasts or myotubes with Trizol. First-strand cDNA was synthesized using RevertAid RT Reverse Transcription Kit (Life Technologies, Carlsbad, CA, USA). Real-time PCR was performed using PerfeCTa SYBR Green FastMix (QuantaBio, USA) in CFX384 Real-time PCR Detection Systems (Bio-Rad). Samples were normalized for expression of GAPDH and analyzed by the 2^−ΔΔCt^ method.

### Statistical analysis

All in vitro experimental data were repeated a minimum of three times. Data are expressed as mean ± the standard error of the mean (S.E.M.). Statistical differences were determined by two-tailed, unpaired Student’s *t* test for two groups or one-way ANOVA with Turkey’s post tests for multiple group comparisons using Prism 8 (Graphpad Software, La Jolla, California). A *P* value < 0.05 was considered to be significant.

## Supplementary Information

**Additional file 1: Table S1.** List of unique proteins identified by hANO5-BioID2. **Table S2.** List of plasmids used in this study. **Figure S1.** Western blot analysis of exogenous expression of human. BVES, POPDC2 and POPDC3 in COS-1 cells. **Figure S2.** Immunofluorescence staining images of COS-1 cells expressing BVES-myc and GFP markers for endosomes and lysosomes. **Figure S3.** Sequence alignment showing the 542bp deletion in the genomic DNA of the E5A8-12 Clone. **Figure S4.** Photographs of WT and Ano5-KO C2C12 cells differentiated for 5 days. Scale bar: 100 μm. **Figure S5.** Differentiation of WT and Bves-KO C2C12 cells differentiated for 5 days. Scale bar: 200 μm. **Figure S6.** Amino acid sequences of Ano5E5-TALEN and Ano5E5ATALEN.

## Data Availability

All relevant data supporting the key findings of this study are available within the article and its Additional file 1 or from the corresponding author upon reasonable request.

## Abbreviations

*ANO5*: Anoctamin 5
*BioID*: Biotin identification
*BVES*: Blood vessel epicardial substance
*CBE*: Cytosine base editing
*CCK8*: Cell counting kit-8
*Co-IP*: Co-immunoprecipitation
*ER*: Endoplasmic reticulum
*FDR*: False discovery rate
*GDD*: Gnathodiaphyseal dysplasia
*gRNA*: Guide RNA
*KO*: Knock out
*LGMDR*: Limb girdle muscular dystrophies recessive
*MMD3*: Miyoshi myopathy type 3
*MyHC*: Myosin heavy chain
*PMR*: Plasma membrane repair
*POPDC*: Popeye domain-containing protein
*TALEN*: Transcription activator like effector nuclease
*WT*: Wild type
