# Supplementary material for: BVES is a novel interactor of ANO5 and regulates myoblast differentiation
Source: Cell Biosci. 2021 Dec 28;11:222. doi: 10.1186/s13578-021-00735-w (PMC8715634; doi:10.1186/s13578-021-00735-w)
Supplement: Supplementary file 1 — Additional file 1: Table S1. List of unique proteins identified by hANO5-BioID2. Table S2. List of plasmids used in this study. Figure S1. Western blot analysis of exogenous expression of human. BVES, POPDC2 and POPDC3 in COS-1 cells. Figure S2. Immunofluorescence staining images of COS-1 cells expressing BVES-myc and GFP markers for endosomes and lysosomes. Figure S3. Sequence alignment showing the 542bp deletion in the genomic DNA of the E5A8-12 Clone. Figure S4. Photographs of WT and Ano5-KO C2C12 cells differentiated for 5 days. Scale bar: 100 μm. Figure S5. Differentiation of WT and Bves-KO C2C12 cells differentiated for 5 days. Scale bar: 200 μm. Figure S6. Amino acid sequences of Ano5E5-TALEN and Ano5E5ATALEN. [file 13578_2021_735_MOESM1_ESM.pdf]

Supplementary Table S1. List of unique proteins identified by hANO5-BioID2.

Accession	Protein Name
Q9QZD9|EIF3I_MOUSE	Eukaryotic translation initiation factor 3 subunit I OS=Mus musculus GN=Eif3i PE=1 SV=1
Q9ES83|POPD1_MOUSE	Blood vessel epicardial substance OS=Mus musculus GN=Bves PE=1 SV=1
Q9ES81|POPD3_MOUSE	Popeye domain-containing protein 3 OS=Mus musculus GN=Popdc3 PE=1 SV=1
Q9EQK5|MVP_MOUSE	Major vault protein OS=Mus musculus GN=Mvp PE=1 SV=4
Q9DC70|NDUS7_MOUSE	NADH dehydrogenase [ubiquinone] iron-sulfur protein 7, mitochondrial OS=Mus musculus GN=Ndufs7 PE=1 SV=1
Q9DBG6|RPN2_MOUSE	Dolichyl-diphosphooligosaccharide--protein glycosyltransferase subunit 2 OS=Mus musculus GN=Rpn2 PE=1 SV=1
Q9D7G0|PRPS1_MOUSE	Ribose-phosphate pyrophosphokinase 1 OS=Mus musculus GN=Prps1 PE=1 SV=4
Q9D773|RM02_MOUSE	39S ribosomal protein L2, mitochondrial OS=Mus musculus GN=Mrpl2 PE=1 SV=1
Q9D2G2|ODO2_MOUSE	Dihydrolipoyllysine-residue succinyltransferase component of 2-oxoglutarate dehydrogenase complex, mitochondrial OS=Mus musculus GN=Dlst PE=1 SV=1
Q9CZW4|ACSL3_MOUSE	Long-chain-fatty-acid--CoA ligase 3 OS=Mus musculus GN=Acsl3 PE=1 SV=2
Q9CPR5|RM15_MOUSE	39S ribosomal protein L15, mitochondrial OS=Mus musculus GN=Mrpl15 PE=1 SV=1
Q99LX5|MMTA2_MOUSE	Multiple myeloma tumor-associated protein 2 homolog OS=Mus musculus GN=Mmtag2 PE=2 SV=1
Q99L43|CDS2_MOUSE	Phosphatidate cytidylyltransferase 2 OS=Mus musculus GN=Cds2 PE=1 SV=1
Q91VH6|MEMO1_MOUSE	Protein MEMO1 OS=Mus musculus GN=Memo1 PE=1 SV=1
Q8VCM8|NCLN_MOUSE	Nicalin OS=Mus musculus GN=Ncln PE=1 SV=2
Q8VBW5-2|BBX_MOUSE	Isoform 2 of HMG box transcription factor BBX OS=Mus musculus GN=Bbx
Q8R010|AIMP2_MOUSE	Aminoacyl tRNA synthase complex-interacting multifunctional protein 2 OS=Mus musculus GN=Aimp2 PE=1 SV=2
Q8K224|NAT10_MOUSE	RNA cytidine acetyltransferase OS=Mus musculus GN=Nat10 PE=1 SV=1
Q8BU33|ILVBL_MOUSE	Acetolactate synthase-like protein OS=Mus musculus GN=Ilvbl PE=1 SV=1
Q8BJW6|EIF2A_MOUSE	Eukaryotic translation initiation factor 2A OS=Mus musculus GN=Eif2a PE=1 SV=2

Q8BHC4|DCAKD_MOUSE	Dephospho-CoA kinase domain-containing protein OS=Mus musculus GN=Dcakd PE=1 SV=1
Q7TMK9|HNRPQ_MOUSE	Heterogeneous nuclear ribonucleoprotein Q OS=Mus musculus GN=Syncrip PE=1 SV=2
Q75UR0|ANO5_MOUSE	Anoctamin-5 OS=Mus musculus GN=Ano5 PE=2 SV=1
Q6ZPE2|MTMR5_MOUSE	Myotubularin-related protein 5 OS=Mus musculus GN=Sbf1 PE=1 SV=2
Q569Z5|DDX46_MOUSE	Probable ATP-dependent RNA helicase DDX46 OS=Mus musculus GN=Ddx46 PE=1 SV=2
Q3UL36|ARGL1_MOUSE	Arginine and glutamate-rich protein 1 OS=Mus musculus GN=Arglu1 PE=1 SV=2
Q3UJD6-2|UBP19_MOUSE	Isoform 2 of Ubiquitin carboxyl-terminal hydrolase 19 OS=Mus musculus GN=Usp19
Q3UDE2|TTL12_MOUSE	Tubulin--tyrosine ligase-like protein 12 OS=Mus musculus GN=Ttll12 PE=1 SV=1
Q3TKT4-2|SMCA4_MOUSE	Isoform 2 of Transcription activator BRG1 OS=Mus musculus GN=Smarca4
P70402|MYBPH_MOUSE	Myosin-binding protein H OS=Mus musculus GN=Mybph PE=2 SV=2
P62814|VATB2_MOUSE	V-type proton ATPase subunit B, brain isoform OS=Mus musculus GN=Atp6v1b2 PE=1 SV=1
P60335|PCBP1_MOUSE	Poly(rC)-binding protein 1 OS=Mus musculus GN=Pcbp1 PE=1 SV=1
P54775|PRS6B_MOUSE	26S proteasome regulatory subunit 6B OS=Mus musculus GN=Psmc4 PE=1 SV=2
P42932|TCPQ_MOUSE	T-complex protein 1 subunit theta OS=Mus musculus GN=Cct8 PE=1 SV=3
P41105|RL28_MOUSE	60S ribosomal protein L28 OS=Mus musculus GN=Rpl28 PE=1 SV=2
P23249|MOV10_MOUSE	Putative helicase MOV-10 OS=Mus musculus GN=Mov10 PE=1 SV=2
P17427|AP2A2_MOUSE	AP-2 complex subunit alpha-2 OS=Mus musculus GN=Ap2a2 PE=1 SV=2
P14873|MAP1B_MOUSE	Microtubule-associated protein 1B OS=Mus musculus GN=Map1b PE=1 SV=2
O88455|DHCR7_MOUSE	7-dehydrocholesterol reductase OS=Mus musculus GN=Dhcr7 PE=1 SV=1
O70475|UGDH_MOUSE	UDP-glucose 6-dehydrogenase OS=Mus musculus GN=Ugdh PE=1 SV=1
O08528|HXK2_MOUSE	Hexokinase-2 OS=Mus musculus GN=Hk2 PE=1 SV=1

Supplementary Table S2. List of plasmids used in this study.

Plasmid	Name	Description
**pYG4010**	plenti-hAno5 WT-BioID2	Lentiviral vector expressing human ANO5 tagged with N-terminal 3xFLAG, C-terminal BioID2. Hygromycin selection
**pYG7012**	pLVX-mAno6-BioID2	Lentiviral vector expressing mouse Ano6 tagged with C-terminal BioID2. Puromycin selection
**pYG7032**	pLVX-BioID2-mMG53	Lentiviral vector expressing mouse MG53 tagged with N-terminal myc and BioID2. Puromycin selection
**pHW_008**	pCDNA3.1-hBVES-Myc	Expression vector for human BVES with C-terminal myc tag
**pHW_009**	pCDNA3.1-hPOPDC2-Myc	Expression vector for human POPDC2 with C-terminal myc tag
**pHW_010**	pCDNA3.1-hPOPDC3-Myc	Expression vector for human POPDC3 with C-terminal myc tag
**pXL-0311**	plenti-Flag-hAno5-WT-intron6-EGFP	Lentiviral vector expressing human ANO5 tagged with N-terminal 3xFLAG, C-terminal GFP. Hygromycin selection
**pXL_893**	plenti-hAno5 1-433aa-ECFP	Lentiviral vector expressing human ANO5 N-terminus 1-433 tagged with N-terminal 3xFLAG and C-terminal ECFP. Hygromycin selection
**pXL-0786**	Flag-hAno5 1-277 aa-EGFP	Expression vector for human ANO5 N-terminus 1-277 tagged with N-terminalFLAG and C-terminal EGFP.
**pXL-0787**	Flag-hAno5 1-150 aa-EGFP	Expression vector for human ANO5 N-terminus 1-150 tagged with N-terminalFLAG and C-terminal EGFP.
**pXL-0788**	Flag-hAno5 1-121 aa-EGFP	Expression vector for human ANO5 N-terminus 1-121 tagged with N-terminalFLAG and C-terminal EGFP.
**pXL-0790**	Flag-hAno5 1-64 aa-EGFP	Expression vector for human ANO5 N-terminus 1-64 tagged with N-terminalFLAG and C-terminal EGFP.
**pHW_019**	pLVX-hBVES-3HA-puro-del 113-171	Lentiviral vector expressing human BVES_del113-171 tagged with C-terminal 3xHA. Puromycin selection
**pYZ1085**	pLVX-hBVES-3HA-puro-del 172-267	Lentiviral vector expressing human BVES_del172-267 tagged with C-terminal 3xHA. Puromycin selection

**pHW_020**	pLVX-hBVES-3HA-puro-truncates-1-267 aa	Lentiviral vector expressing human BVES_1-267 tagged with C-terminal 3xHA. Puromycin selection
**pXL-0057**	pLVX-mcherry-mAno5	Lentiviral vector expressing mouse Ano5 fused with mCherry. Puromycin selection
**pXL-0111**	pEGFP-E2A-TAL6-mAno5-E5	Expression vector for mouse Ano5 E5-TALEN pair
**pXL-0112**	pEGFP-E2A-TAL6-mAno5-E5A	Expression vector for mouse Ano5 E5A-TALEN pair
**pXL-0569**	pCMV_AncBE4max	Addgene #112094
**pYZ1097**	pCMV-AncBE4-GFP	Expression vector for AncBE4-2A-GFP fusion
**pYZ1083**	pLenti-mBVES-gRNA	Lentiviral vector expressing mouse BVES targeting guide RNA. Zeocin selection
**pHW_004**	pEGFP-C1-EEA1	Addgene #42307
**pHW_005**	pEGFP-C1-Rab7	Addgene #12605
**pHW_006**	pEGFP-C1-Rab11	Addgene #12674
**pHW_007**	pEGFP-N1-LAMP1	Addgene #34831

Supplementary Figure S1. Western blot analysis of exogenous expression of human BVES, POPDC2 and POPDC3 in COS-1 cells.

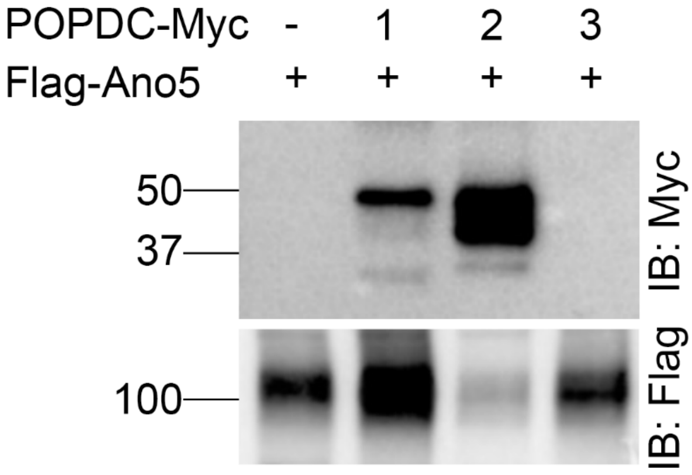

Supplementary Figure S2. Immunofluorescence staining images of COS-1 cells expressing BVES-myc and GFP markers for endosomes and lysosomes.

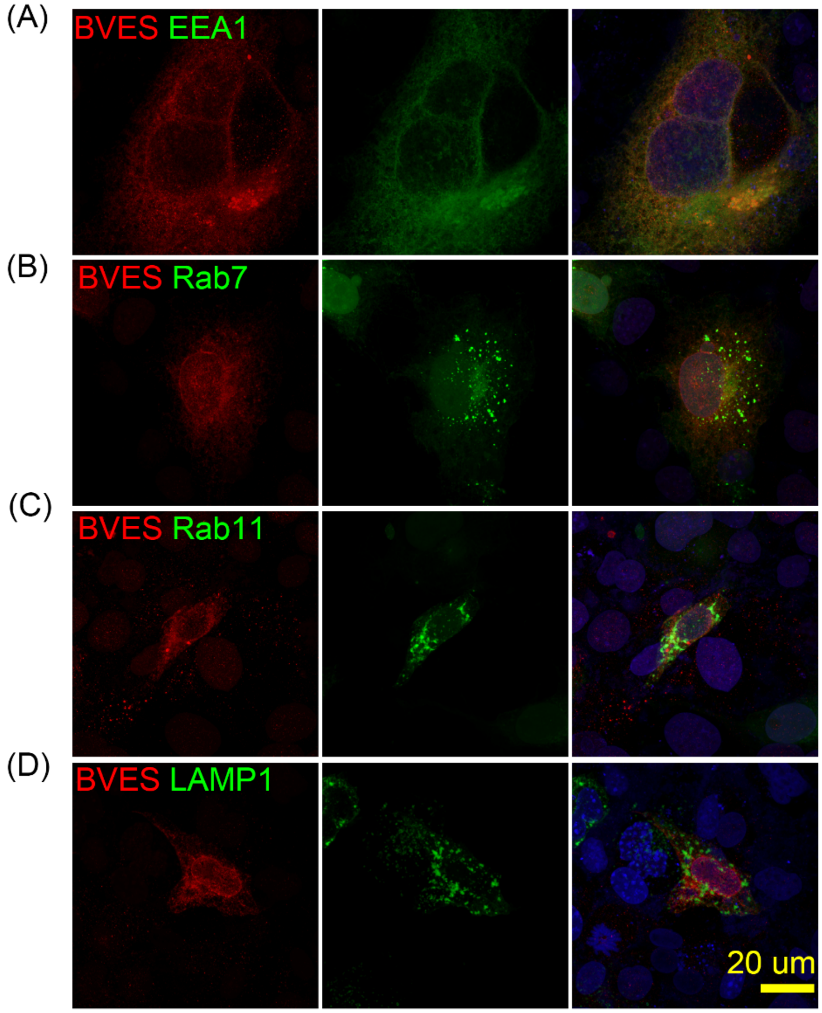

Supplementary Figure S3. Sequence alignment showing the 542bp deletion in the genomic DNA of the E5A8-12 Clone.

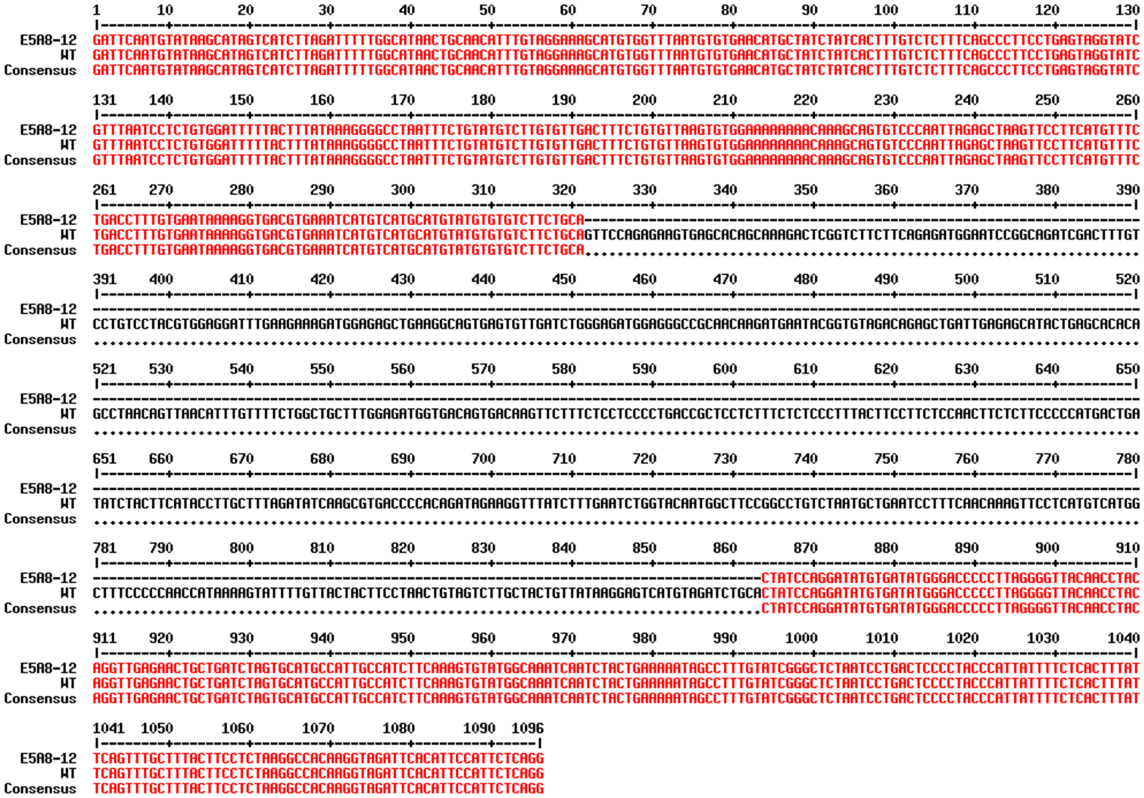

Supplementary Figure S4. Photographs of WT and *Ano5*-KO C2C12 cells differentiated for 5 days. Scale bar: 100 µm.

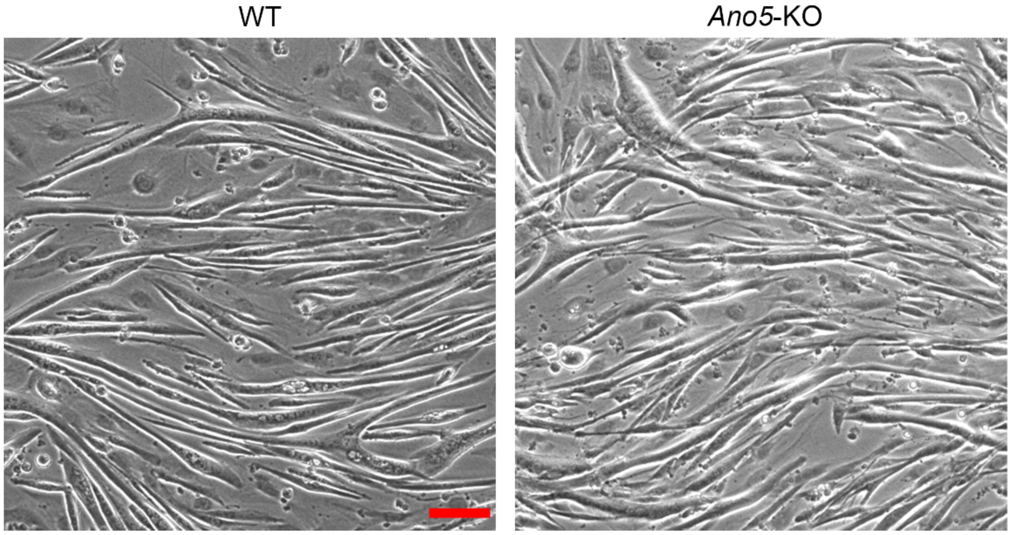

Supplementary Figure S5. Differentiation of WT and *Bves*-KO C2C12 cells differentiated for 5 days. Scale bar: 200 µm.

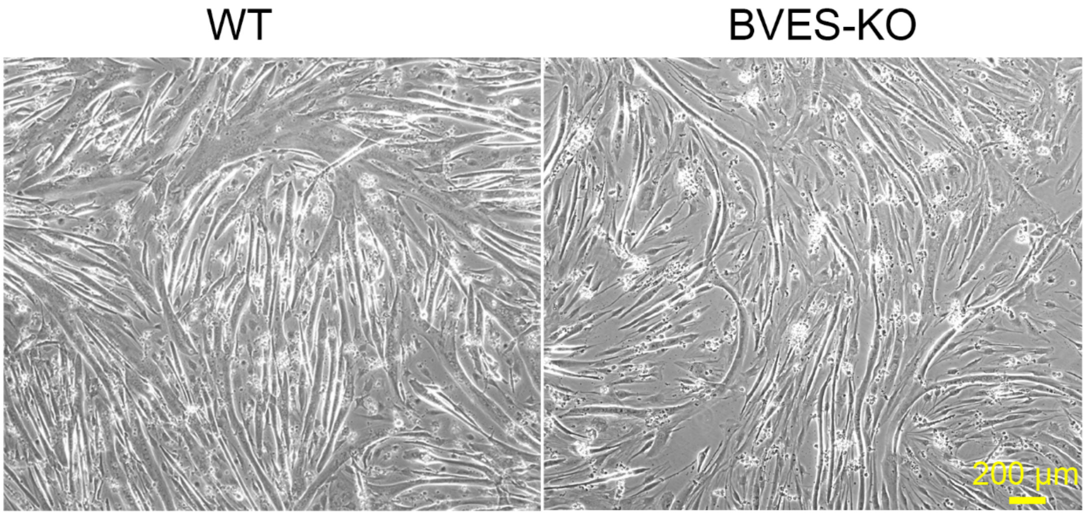

Supplementary Figure S6. Amino acid sequences of Ano5E5-TALEN and Ano5E5A-TALEN.

>Ano5E5-TALEN

MDYKDHDGDYKDHDIDYKDDDDKMAPKKKRKVGIHGVPMVDLRTLGYSQQQQEKIKP
KVRSTVAQHHEALVGHGFTHAHIVALSQHPAALGTVAVTYQHIITALPEATHEDIVGVG
KQWSGARALEALLTDAGELRGPPLQLDTGQLVKIAKRGGVTAMEAVHASRNALTGAP

LNLTPDQVVAIASHDGGKQALETVQRLLPVLCQDHGLTPDQVVAIASNNGGKQALETV
QRLLPVLCQDHGLTPDQVVAIASNIGGKQALETVQRLLPVLCQDHGLTPDQVVAIASHD
GGKQALETVQRLLPVLCQDHGLTPDQVVAIASNGGGKQALETVQRLLPVLCQDHGLT
PDQVVAIASNGGGKQALETVQRLLPVLCQDHGLTPDQVVAIASNGGGKQALETVQRLL
PVLCQDHGLTPDQVVAIASNNGGKQALETVQRLLPVLCQDHGLTPDQVVAIASNGGG
KQALETVQRLLPVLCQDHGLTPDQVVAIASHDGGKQALETVQRLLPVLCQDHGLTPDQ
VVAIASHDGGKQALETVQRLLPVLCQDHGLTPDQVVAIASNGGGKQALETVQRLLPVL
CQDHGLTPDQVVAIASNNGGKQALETVQRLLPVLCQDHGLTPDQVVAIASNGGGKQA
LETVQRLLPVLCQDHGLTPDQVVAIASHDGGKQALETVQRLLPVLCQDHGLTPDQVVA
IASHDGGKQALETVQRLLPVLCQDHGLTPDQVVAIASNGGGKQALESIVAQLSRPDPA
LAALTNDHLVALACLGGRPAMDAVKKGLPHAPELIRRVNRRIGERTSHRVAGSQLVKS
ELEEKKSELRHKLKYVPHEYIELIEIARNPTQDRILEMKVMEFFMKVYGYRGEHLGGSR
KPDGAIYTVGSPIDYGVIVDTKAYSGGYNLPIGQADEMERYVEENQTRDKHLNPNEW
WKVYPSSVTEFKFLFVSGHFKGNYKAQLTRLNHITNCNGAVLSVEELLIGGEMIKAGTL
TLEEVRRKFNNGEINFRSGGGEGRGSLLTCGDVEENPGPRMDYKDHDGDYKDHDIDY
KDDDDKMAPKKKRKVGIHGVPMVDLRTLGYSQQQQEKIKPKVRSTVAQHHEALVGHG
FTHAHIVALSQHPAALGTVAVTYQHIITALPEATHEDIVGVGKQWSGARALEALLTDAG
ELRGPPLQLDTGQLVKIAKRGGVTAMEAVHASRNALTGAPLNLTPDQVVAIASNNGGK
QALETVQRLLPVLCQDHGLTPDQVVAIASHDGGKQALETVQRLLPVLCQDHGLTPDQV
VAIASHDGGKQALETVQRLLPVLCQDHGLTPDQVVAIASNGGGKQALETVQRLLPVLC
QDHGLTPDQVVAIASNGGGKQALETVQRLLPVLCQDHGLTPDQVVAIASHDGGKQAL
ETVQRLLPVLCQDHGLTPDQVVAIASNIGGKQALETVQRLLPVLCQDHGLTPDQVVAIA
SNNGGKQALETVQRLLPVLCQDHGLTPDQVVAIASHDGGKQALETVQRLLPVLCQDH
GLTPDQVVAIASNGGGKQALETVQRLLPVLCQDHGLTPDQVVAIASHDGGKQALETVQ
RLLPVLCQDHGLTPDQVVAIASNGGGKQALETVQRLLPVLCQDHGLTPDQVVAIASHD
GGKQALETVQRLLPVLCQDHGLTPDQVVAIASHDGGKQALETVQRLLPVLCQDHGLT
PDQVVAIASNIGGKQALETVQRLLPVLCQDHGLTPDQVVAIASNGGGKQALETVQRLL
PVLCQDHGLTPDQVVAIASHDGGKQALETVQRLLPVLCQDHGLTPDQVVAIASNGGG
KQALETVQRLLPVLCQDHGLTPDQVVAIASNGGGKQALESIVAQLSRPDPALAALTND
HLVALACLGGRPAMDAVKKGLPHAPELIRRVNRRIGERTSHRVAGSQLVKSELEEKKS
ELRHKLKYVPHEYIELIEIARNPTQDRILEMKVMEFFMKVYGYRGEHLGGSRKPDGAIY
TVGSPIDYGVIVDTKAYSGGYNLPIGQADEMQRYVKENQTRNKHINPNEWWKVYPSS
VTEFKFLFVSGHFKGNYKAQLTRLNRKTNCNGAVLSVEELLIGGEMIKAGTLTLEEVRR
KFNNGEINF

>Ano5E5A-TALEN

MDYKDHDGDYKDHDIDYKDDDDKMAPKKKRKVGIHGVPMVDLRTLGYSQQQQEKIKP
KVRSTVAQHHEALVGHGFTHAHIVALSQHPAALGTVAVTYQHIITALPEATHEDIVGVG
KQWSGARALEALLTDAGELRGPPLQLDTGQLVKIAKRGGVTAMEAVHASRNALTGAP
LNLTPDQVVAIASHDGGKQALETVQRLLPVLCQDHGLTPDQVVAIASHDGGKQALETV
QRLLPVLCQDHGLTPDQVVAIASNIGGKQALETVQRLLPVLCQDHGLTPDQVVAIASNN
GGKQALETVQRLLPVLCQDHGLTPDQVVAIASNIGGKQALETVQRLLPVLCQDHGLTP
DQVVAIASNNGGKQALETVQRLLPVLCQDHGLTPDQVVAIASNIGGKQALETVQRLLP

VLCQDHGLTPDQVVAIASNIGGKQALETVQRLLPVLCQDHGLTPDQVVAIASNNGGKQ
ALETVQRLLPVLCQDHGLTPDQVVAIASNGGGKQALETVQRLLPVLCQDHGLTPDQVV
AIASNNGGKQALETVQRLLPVLCQDHGLTPDQVVAIASNIGGKQALETVQRLLPVLCQD
HGLTPDQVVAIASNNGGKQALETVQRLLPVLCQDHGLTPDQVVAIASHDGGKQALETV
QRPVQRLLPVLCQDHGLTPDQVVAIASNIGGKQALESIVAQLSRPDPALAALTNDHLVA
LACLGGRPAMDAVKKGLPHAPELIRRVNRRIGERTSHRVAGSQLVKSELEEKKSELRH
KLKYVPHEYIELIEIARNPTQDRILEMKVMEFFMKVYGYRGEHLGGSRKPDGAIYTVGS
PIDYGVIVDTKAYSGGYNLPIGQADEMERYVEENQTRDKHLNPNEWWKVYPSSVTEF
KFLFVSGHFKGNYKAQLTRLNHITNCNGAVLSVEELLIGGEMIKAGTLTLEEVRRKFNN
GEINFRSGGGEGRGSLLTCGDVEENPGPRMDYKDHDGDYKDHDIDYKDDDDKMAPK
KKRKVGIHGVPMVDLRTLGYSQQQQEKIKPKVRSTVAQHHEALVGHGFTHAHIVALSQ
HPAALGTVAVTYQHIITALPEATHEDIVGVGKQWSGARALEALLTDAGELRGPPLQLDT
GQLVKIAKRGGVTAMEAVHASRNALTGAPLNLTPDQVVAIASNGGGKQALETVQRLLP
VLCQDHGLTPDQVVAIASHDGGKQALETVQRLLPVLCQDHGLTPDQVVAIASHDGGK
QALETVQRLLPVLCQDHGLTPDQVVAIASNIGGKQALETVQRLLPVLCQDHGLTPDQV
VAIASNGGGKQALETVQRLLPVLCQDHGLTPDQVVAIASHDGGKQALETVQRLLPVLC
QDHGLTPDQVVAIASNGGGKQALETVQRLLPVLCQDHGLTPDQVVAIASHDGGKQAL
ETVQRLLPVLCQDHGLTPDQVVAIASNGGGKQALETVQRLLPVLCQDHGLTPDQVVAI
ASNNGGKQALETVQRLLPVLCQDHGLTPDQVVAIASNIGGKQALETVQRLLPVLCQDH
GLTPDQVVAIASNIGGKQALETVQRLLPVLCQDHGLTPDQVVAIASNNGGKQALETVQ
RLLPVLCQDHGLTPDQVVAIASNIGGKQALETVQRPVQRLLPVLCQDHGLTPDQVVAIA
SNIGGKQALESIVAQLSRPDPALAALTNDHLVALACLGGRPAMDAVKKGLPHAPELIRR
VNRRIGERTSHRVAGSQLVKSELEEKKSELRHKLKYVPHEYIELIEIARNPTQDRILEMK
VMEFFMKVYGYRGEHLGGSRKPDGAIYTVGSPIDYGVIVDTKAYSGGYNLPIGQADEM
QRYVKENQTRNKHINPNEWWKVYPSSVTEFKFLFVSGHFKGNYKAQLTRLNRKTNCN
GAVLSVEELLIGGEMIKAGTLTLEEVRRKFNNGEINF
